# Isolated *Lactobacillus fermentum* Ab.RS22 from traditional dairy products inhibits HeLa cervical cancer cell proliferation and modulates apoptosis by the PTEN-Akt pathway

**DOI:** 10.22038/IJBMS.2023.72825.15846

**Published:** 2024

**Authors:** Abbas Asoudeh-Fard, Mitra Salehi, Dariush Ilghari, Asghar Parsaei, Peyman Heydarian, Hossein Piri

**Affiliations:** 1 INSERM U1148, Laboratory for Vascular Translation Science (LVTS), Cardiovascular Bioengineering, University Sorbonne Paris North, Paris, France; 2 Research Center for Pharmaceutical Nanotechnology, Tabriz University of Medical Sciences, Tabriz, Iran; 3 Clinical Research Development Unit, Booalisina Hospital, Qazvin University of Medical Sciences, Qazvin, Iran; 4 Student Research Committee, Qazvin University of Medical Sciences, Qazvin, Iran; 5 Clinical Pharmacist, Baylor Scott & White Medical Center – Lakeway 100 Medical Pkwy, Lakeway, TX 78738; 6 Rayan Novin Pajoohan Pras, Biotechnology Company, Biotechnology Incubator, Shiraz University of Medical Sciences, Shiraz, Iran; 7 Medical Microbiology Research Center, Qazvin University of Medical Sciences, Qazvin, Iran; 8 Department of Medical Parasitology and Mycology, School of Medicine, Qazvin University of Medical Sciences, Qazvin, Iran; 9 Department of Biochemistry and Genetics, School of Medicine, Qazvin University of Medical Sciences, Qazvin, Iran

**Keywords:** Apoptosis, Lactobacillus, Microbiology, Probiotics, PTEN protein, Tumor suppressor protein, p53

## Abstract

**Objective(s):**

It is worthwhile to note that, some probiotics such as *Lactobacilli* and *Bifidobacteria* isolated from dairy products have significant therapeutic effects against cancer cells. Here, we evaluated anti-proliferation and the apoptotic effects of isolated *Lactobacillus fermentum* Ab.RS22 from traditional dairy products on the HeLa cervical cancer cells *in vitro.*

**Materials and Methods:**

The viability of treated HeLa cells with supernatant of *Lactobacillus* in 0.5, 0.75, 1, 1.5, and 2 ng/ml concentrations, and IC_50_ values were detected by tetrazolium bromide. The *L. fermentum *Ab.RS22-induced cell death by ﬂow cytometry was confirmed through evaluation of the expression of *caspase-3*, *P53*, *PTEN*, and *AKT* genes by quantitative reverse transcription-polymerase chain reactions (qRT-PCR).

**Results:**

Most cytotoxicity effects of *Lactobacillus* on HeLa cells were detected in 2 ng/ml at 24 hr (*P*<0.01); also, the IC_50_ value was measured as 1.5 ng/ml. The findings of the flow cytometry assay showed that *L. fermentum *Ab.RS22 in 1.5 ng/ml concentration at 24 hr increased the percentage of both apoptosis and necrosis cells. Lactobacillus-induced cell death was verified through results of Real-time PCR; where expression of* caspase-3*, *P53*, and *PTEN* genes was increased (*P*<0.01), and also expression of *AKT* gene (anti-apoptotic) was decreased (*P*<0.05).

**Conclusion:**

Our findings showed that *L. fermentum* Ab.RS22 could dose-dependently inhibit the proliferation of the HeLa cells. Its apoptotic effect was confirmed via modulating *PTEN/p53*/*Akt* gene expression and activation of the caspase-3 mediated apoptosis pathway. Therefore, *L. fermentum *Ab.RS22 can be considered a valuable anticancer candidate against cervical cancer progression in subsequent studies.

## Introduction

Currently, cervical cancer affects many females and is the fourth most common cancer worldwide with 604,127 new cases and 341,831 deaths in 2020 (1). Human papillomavirus (HR-HPV) and herpes simplex virus type-2 (HSV2) infections (sexually transmitted infections) as well as obesity, and early menopause are high-risk factors in developing cervical cancer. Prophylactic screening including polymerase chain reaction (PCR)-based tests and smear testing are worthwhile screenings for early recognition of HPV infection (2). Despite the use of prophylactic screening and new treatment methods such as immunotherapy, the mortality rate is increasing. Hence, identifying new treatment approaches and effective preventative vaccines is highly demanded (3).

Living microbes (microflora) that live in the vagina have a dynamic balance state in healthy women; these microbes play a critical role in the normal functions of the immune system and sex hormone balance (4, 5). Frąszczak *et al*. have reported that chronic inflammation results in tumorigenesis; which is a key inducer of chronic inflammation in cervical cancer and is a disruption of the homeostasis of the sexual microbiome in the vagina (microbiota inflammation) (6). Contraception, sex life, dietary habits, and sanitary conditions of the living area are affected by the hemostasis of vaginal microbiota. Based on the worthwhile roles of the microbiome in vaginal health, researchers, by sequencing of *16S rRNA* vaginal microbiome gene, determined the microbiome profile in a woman’s reproductive tract (4). One of the most protective and safe probiotics in the reproductive tract of the female is lactobacillus. The protective effects of *Lactobacillus *in cervical cancer are correlated with antimicrobial function (anaerobic bacteria) and anti-tumor activity through the secretion of bacteriocins and clearance of the HR-HPV (7). Based on the low side effects and availability of *Lactobacillus*, many researchers have been paid to study these probiotics and their metabolites in the synthesis of HPV vaccine in immunotherapy and its possible treatment effects in cervical cancer before application in clinical studies (8). 

Inhibiting tumor progression through a proper dose of probiotics in various cancers has been researched so far. For instance, a study demonstrated that *Lactobacillus rhamnosus* exhibited anti-inflammatory and apoptotic effects in colon cancer (Dawley rats model) via modulating the expression of *Bcl-2*, *p53*, *BAX*, *Caspase- 3*, Vascular Endothelial Growth Factor A (*VEGFa*), and Tumor necrosis factor alpha (TNF-α) genes (9). Modulation of the expression of oncogenes and tumor suppressor genes including the *Bcl-2* family, caspase enzymes, phosphatase and tensin homolog (*PTEN*), and *p53* are beneficial markers for monitoring, diagnosis, and targeted treatment of metastatic cancer (10, 11). Accordingly, this study focused on the pre-apoptotic effects of isolated *L. fermentum *Ab.RS22 from the traditional dairy products on HeLa human cervical cancer cells through cellular and molecular assessments *in vitro*. As the molecular assessment, mRNA expression of *caspase-3*, *P53*, *PTEN*, and *AKT* genes was evaluated by quantitative reverse transcription-polymerase chain reactions (qRT-PCR). Moreover, a flow cytometry assay was used for monitoring the percentage of apoptotic and necrotic cells and for reconfirming the results of the molecular assessment.

## Materials and Methods

Dulbecco’s Modified Eagle Medium (DMEM) /High Glucose was obtained from Capricorn Co. (Cat. No. DMEM-HA, Germany). Respectively, Penicillin-Streptomycin antibiotics (10,000 units penicillin and 10 mg streptomycin), Thiazolyl Blue Tetrazolium Bromide (MTT), and Trypsin – EDTA (1X) were purchased from Sigma-Aldrich Co. (Poole, UK). Fetal Bovine Serum (Heat Inactivated Qualified FBS) and Dimethyl Sulfoxide (DMSO) were from Thermo Fisher Scientific. The extraction DNA Kit was obtained from Sinaclon Co. (Tehran, Iran). The total RNA Extraction Kit was from Parstous Co. (Tehran, Iran). TruScript First Strand cDNA Synthesis Kit (Cat. No. 54420) was purchased from Norgen, Canada. Master Mix Real-Time PCR (2X) (Cat. No. 28340) was obtained from (Norgen, Canada), also Taq DNA Polymerase 2x Master Mix (Cat. No. A190303) was from (Ampliqon, Denmark). Microbial Culture Media – MRS Broth (Cat. No. 1106610500) was purchased from Merck CO. (Germany). Apoptosis detection Kit (FITC-Annexin /Pi) was obtained from Thermo Fisher Scientific (Cat. No. BMS500FI-100). Primers (*16SrRNA*, *caspase-3*, *P53*, *PTEN*, *AKT*, and *GAPDH*) were taken from Sinaclon Co. (Tehran, Iran). 

***Microbial strain and molecular identification***

Based on a study, *L. fermentum *was isolated from dairy products of Guilan villages in Iran (12). Briefly, the isolated *L.* *fermentum* strains were cultured in the Man-Rogosa-Sharpe broth (MRS) and incubated at 30 °C for 48 hr. Subsequently, fresh *L. fermentum* suspensions were isolated from MRS by centrifugations at (12000 g, 10 min, and 4 °C) conditions; then the pellet was washed with Tris-HCI butter twice at (pH =7.0 and pH= 8.0, respectively) and incubated at 37 °C for 2 hr to remove the dead bacteria. Finally, the collected pellet was re-suspended in sterile phosphate-buffered saline to prepare our dilutions (0.5, 0.75, 1, 1.5, and 2 ng/ml). Molecular identification of Ab.RS22 strain of* L. fermentum *was conducted through 16S rRNA gene sequencing after DNA extraction. The sequence of 16S rRNA primer is shown in Table 1. In the end, agarose gel (1%) was used to confirm the PCR product amplification. 

***Cell culture and treatment***

The frozen human cervical cancer cell (HeLa cell) (ATCC^®^CRL-12401) was purchased from the Pasteur Institute (Tehran, Iran). The HeLa cells were cultured in supplemented DMEM with FBS and pen-strep antibiotics at 1.0×10^4^ cells/cm^2^ density in a humidified incubator with 5% CO_2_ and 37 °C conditions (Thermo, USA). Firstly, during 24 hr bacteria were cultured in MRS Broth (Microbial Culture Media), and subsequently, dead bacteria were harvested and their pallets washed with PBS. In the next step, after overnight incubation (confluency of cultured cells: 40%–50%), cells were treated (4–5 hr) with the media multiplex containing metabolites and signaling molecules of *L. fermentum* and cells. Then, the conditioned medium from the previous step was filtered and treated with HeLa cells in* (*OD600: 0.5, 0.75, 1, 1.5, and 2 ng/ml) at 24 hr (13). 

***Cell viability assay***

To assess the cytotoxicity and determine the IC_50_ value of* L. fermentum *Ab.RS22 in HeLa cells, the MTT assay was used, and results were statically analyzed through Graph Pad Prism software. After overnight incubation of cells in a 96-well plate (2× 10^3^ cells/well), DMEM medium was pulled out and cells were treated with (OD600: 0.5, 0.75, 1, 1.5, and 2 ng/ml) conditioned medium for 24 hr in a humidified incubator. Afterward, 20 µl of MTT solution with 5 mg/ml concentration was added to flat bottom wells in 3 hr. After observing the formazan precipitate in wells, DMSO solution (150 µl) was added to wells (14). Finally, the absorbance values of each well (samples and control groups) were measured by an ELISA Plate Reader (Hyperion, Germany). The IC_50_ value was treated with cells in the following methods.

***Flow cytometry assay***

The detection of cell apoptosis was quantified through the fluorescein isothiocyanate annexin v propidium iodide (FITC-Annexin/Pi) kit and BD FACS Calibur flow cytometry (BD Biosciences, San Jose, CA, USA). In short, HeLa cells (5 × 10^5^ cells/ well) were incubated with the IC_50_ value of *L. fermentum *Ab.RS22 overnight. Then, the DMEM medium was pulled out and cells were washed with PBS. According to the instructions of the mentioned kit, ﬂoated and adherent cells in wells were centrifuged (5 min, 1500 rpm) for the cell pallets formation; then pallets were washed with cold PBS and binding buﬀer (300 µl). Finally, FITC-Annexin (2 µl, 15min) and Pi (1 µl) were added to samples, respectively (15). 

***RNA extraction, cDNA synthesis, and qRT-PCR***

Assessment of the mRNA expression of *caspase-3*, *P53*, *PTEN*, and *AKT* genes was performed through qRT-PCR (Mic Real-Time PCR, Switzerland). Firstly, RNA was extracted based on the Total RNA Extraction Kit of the treated and untreated cells (control group) with IC_50_ value of *L. fermentum *Ab.RS22 overnight. Subsequently, cDNA synthesis of extracted RNA was carried out via Moloney Murine Leukemia Virus Reverse Transcriptase, based on cDNA synthesis kit instruction (Norgen, Canada). The qRT-PCR was performed via Norgen SYBR Green qRT- PCR Master Mix, cDNA, and caspase-3, P53, PTEN, and AKT primers; sequences of targeted genes primer (*caspase-3*, *P53*, *PTEN*, and *AKT*) and house-keeping gene primer Glyceraldehyde 3-phosphate dehydrogenase (*GAPDH*) have been mentioned in Table 1. Analysis of the results was measured based on the pfaff method (2^–ΔΔCT^); standardization of the obtained cyclin threshold (CT) was accomplished through internal reference gene (*GAPDH*) (13). The cyclin amplification condition is shown in Table 2.

***Statistics***

Statistical differences between groups (control versus samples) in all measurements were analyzed via utilization of one-way analysis of variance (ANOVA) followed by Tukey’s examinations; data were considered statistically significant at *P*-values less than 0.05* and 0.01**. The expression of targeted genes and housekeeping genes was analyzed via the SPSS software package (version 26; SPSS Inc, USA), and their figures were drawn by version 9.1.0.221 GraphPad Prism software. The experiments were performed in duplicate.

## Results

***Molecular identification of the isolated bacterium***

**Figure 1:**
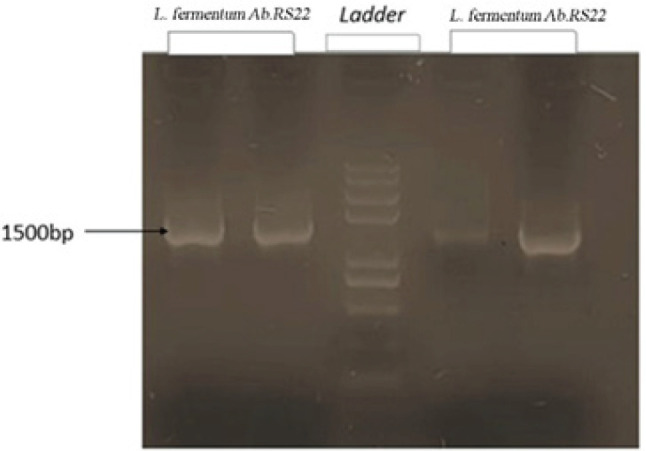
Identifcation of 16srRNA gene of *Lactobacillus fermentum* on agarose gel electrophoresis (1%)

Molecular identification of isolated bacterium was performed by amplification of *16S rRNA* gene through PCR and specific primers. According to Figure 1, a 1500 bp band on the electrophoresis gel (%1) has been shown. Blasting this sequenced *16S rRNA* gene in the National Center for Biotechnology Information (NCBI gene database) showed that the isolated bacterium in our study is considered *L. fermentum *Ab.RS22. The sequence of the bacterium was submitted to the NCBI gene database with the accession number: ON908679.

***Cell viability assay***

**Figure 2:**
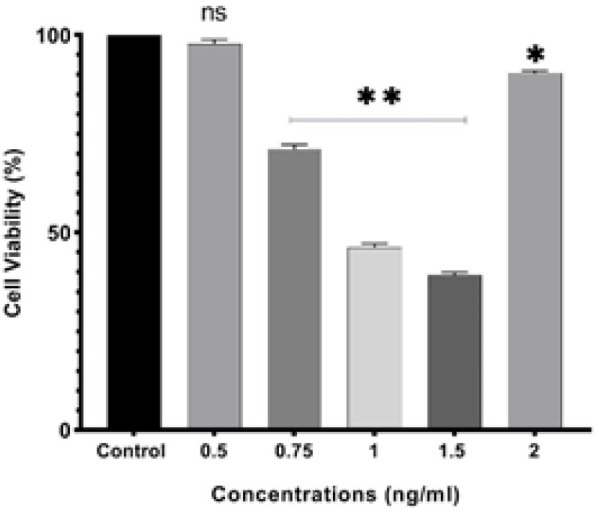
Evaluation of the cytotoxicity of conditioned-medium of *Lactobacillus fermentum *Ab.RS22 in (OD600: 0.5, 0.75, 1, 1.5, and 2 ng/ml) at 24 hr on HeLa cells by MTT assay

In our study, the inhibitory effects of conditioned-medium of *L. fermentum *Ab.RS22 in (OD600: 0.5, 0.75, 1, 1.5, and 2 ng/ml) at 24 hr on the growth of HeLa cells were evaluated through MTT assay. According to Figure 2, it was detected that conditioned-medium of *L. fermentum *Ab.RS22 in (OD600: 1, 1.5, and 2 ng/ml) at 24 hr significantly diminished the viability of HeLa cells in a dose-dependent manner, and the IC_50_ value was determined (OD600: 1.5 ng/ml). Further results indicated that OD600: 0.5, 0.75 ng/ml at 24 hr increased the HeLa cells’ viability compared to untreated cells (control group). It is worthwhile to note that, a (OD600: 1.5) of conditioned-medium of *L. fermentum *Ab.RS22 was selected for treatment with HeLa cells in apoptosis assessment associated with flow cytometry and qRT-PCR methods.

***Cell death assessment of*** ***HeLa cells by flow cytometry ***

**Figure 3:**
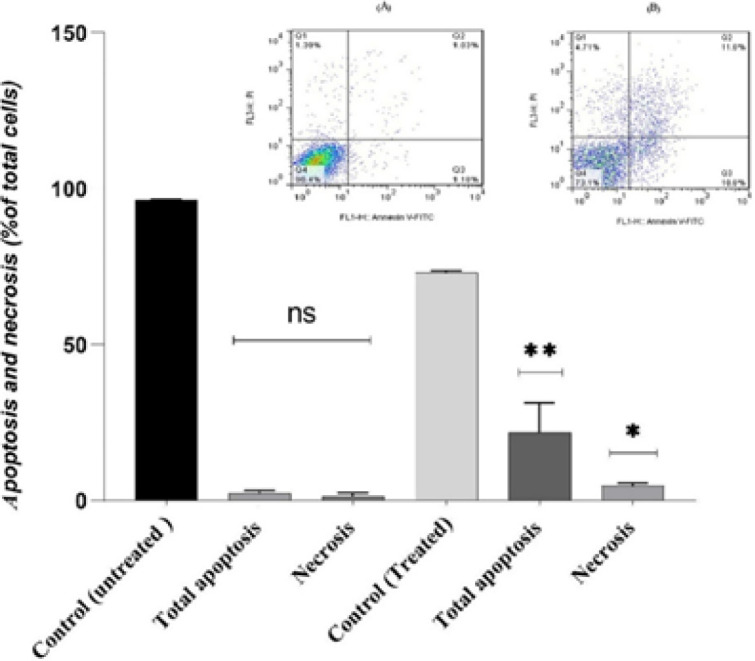
Evaluation of the pre-apoptotic effect of conditioned-medium of *Lactobacillus fermentum *Ab.RS22 on IC_50_ value at 24 hr on HeLa cells by flow cytometry

To this end, we examined the induction of cell death in treated HeLa cells with IC_50_ value of conditioned-medium of *L. fermentum *Ab.RS22 by FITC-Annexin/Pi staining. Overall, the percentage of apoptotic (FITC-Annexin staining) and necrotic (Pi staining) cells in treated HeLa cells increased more than in the control group. According to Figure 3, conditioned-medium of *L. fermentum *Ab.RS22 induced cell apoptosis on HeLa cells by increasing the number of cells in late apoptosis from 1.03% in the control group to 11.6%, and also early apoptosis from 1.18% in the control group to 10.6% in 24 hr. 

***Expression of caspase-3, P53, PTEN, and AKT genes***

**Figure 4:**
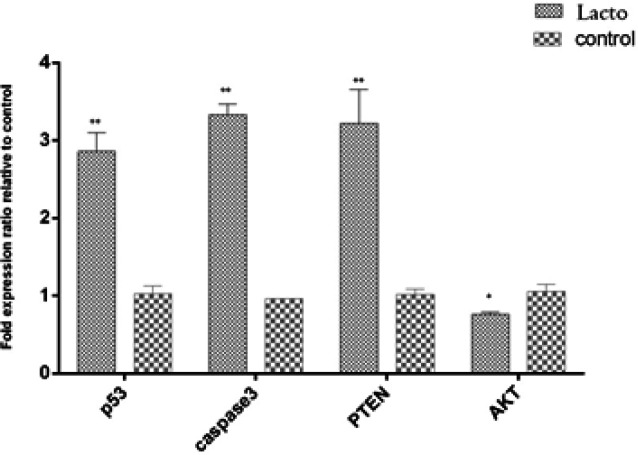
Pre-apoptotic eﬀects of conditioned-medium of *Lactobacillus fermentum *Ab.RS22 (IC_50_ value) on HeLa cells at 24 hr through assessment of the expression of *caspase-3*,* P53*, *PTEN*, and* AKT* genes by qRT-PCR. *P*<0.05*: significant diﬀerence - *P*<0.01**: highly significant diﬀerence

To evaluate the apoptosis-inducing effect of conditioned-medium of *L. fermentum *Ab.RS22 (IC_50_ value) on HeLa cells at 24 hr, mRNA expression of some pro-apoptotic and anti-apoptotic genes including *caspase-3*, *P53*, *PTEN*, and *AKT* was determined through qRT-PCR. As shown in Figure 4, conditioned-medium of *L. fermentum *Ab.RS22 through simultaneous effect in down-regulation of *AKT* gene (*P*<0.05) and up-regulation of *caspase-3*, *P53*, *PTEN* (*P*<0.01) could be regarded as a biological candidate for induction of apoptosis signaling pathways in HeLa cells *in vitro*. 

**Table 1:** Sequences of the primer pairs of internal reference gene and targeted genes for qRT-PCR. (F, forward; R, reverse)

Primer	Gene
**Ab.L. F: 5' AGAGTTTGATCCTGGCTCAG 3'** **Ab.L.R : 5' CTAGTACCAAGGCATTCACC 3'**	**16SrRNA**
**F: 5' AAGCTCATTTCCTGGTATGACAACG 3'** **R: 5' TCTTCCTCTTGTGCTCTTGCTGG 3'**	**GAPDH**
**F: 5- TCCCAGTCAGAGGCGCTATG -3** **R:5- CACAAACTGAGGATTGCAAG -3**	**PTEN**
**F: 5' ACTCTTTCCAGACCCACGAC3'** **R: 5' CTCAAATGCACCCGAGAAAT 3'**	**AKT**
**F: 5' TGCGTGTGGAGTATTTGGATG3'** **R: 5' TGGTACAGTCAGAGCCAACCTC3'**	**P53**
**F: 5′- TGCCTGTAACTTGAGAGTAGATGG -3′** **R: 5′- CTTCACTTTCTTACTTGGCGATGG -3′**	**Caspase-3**

**Table 2:** Cyclin amplification condition for molecular assessment by qRT-PCR

Time	Temperature	Cycle	Stage
**3 min**	95 °C	1	**Initial denaturation**
**10 sec**	95 °C	40	**Stage1 (Denature)**
**20 sec**	52 °C		**Stage2 (Anneal)**
**20 sec**	72 C		**Stage3 (Extend)**

Treated HeLa cells with conditioned medium of *L. fermentum *Ab.RS22. *P*<0.05*: significant diﬀerence - *P*<0.01**: highly significant diﬀerence- ns: not significant

A) control group; B) Treated HeLa cells with a conditioned medium of *Lactobacillus fermentum *Ab.RS22 (1.5 ng/ml). *P*<0.05*: significant diﬀerence - *P*<0.01**: highly significant diﬀerence

ns: not significant

## Discussion

The progression of cervical cancer is related to the vaginal environment and genetic factors. So far, growing research has described the considerable roles of vaginal bacteria (vaginal microbiota) in the reproductive tract health. Among vaginal microbiota, *Lactobacillus *is the dominant microbe in a healthy vagina, which shows suitable defense roles against adhesion and growth of pathogenic microorganisms through the production of numerous metabolic agents such as peptides and lactic acid in the vagina (16). Probiotics were introduced by the World Health Organization (WHO) as live microorganisms, which are composed of *Streptococci*, *Lactobacilli*, and *Bifidobacteria* bacteria. Probiotics by owing vital properties such as bioavailability, cost-effectiveness, and safety are widely used in the reinforcement of the immune system and diminish the inflammatory state in disorders such as allergy, diabetes, and cancerous cells (17). 

According to the beneficial effects of probiotics in human metabolism through the production of essential agents such as specific enzymes in humans, the anticancer activity of probiotics has been reported in numerous previous studies. Recent reports have suggested that symbiotic foods are a modulator of immunological response to inhibit carcinogenesis in cervical cancer. For instance, findings of the research of De Loera Rodríguez *et al*. demonstrated that consumption of symbiotic foods containing *Lactobacilli* and *Bifidobacteria* significantly diminished nausea and vomiting symptoms in patients with CC (16). In addition, results from a meta-analysis study revealed that the consumption of probiotics in a patient with CC could reduce abdominal pain and the severity of radiotherapy-induced diarrhea (RID) in these patients (18). Induced cervical cancer by papillomavirus is estimated as the second cause of this cancer globally. Therapeutic effects of lactobacillus on cervical cancer in HPV-associated tumors in C57BL/6 mice were conducted in 2022, later research showed that consumption of *lactobacillus casei* reinforced the immune responses and diminished the cervical tumor progression (19). Similarly, a study reported the antitumor activity of *Bifidobacterium bifidum* in the progression of HPV-associated tumors of cervical cancer in C57BL/6 mice in which the results have shown that oral administration of these probiotics could significantly modulate the immune system and reduce tumor growth *in vivo* (20). 

Despite the many studies that have reported anticancer eﬀects of *Lactobacilli* in cancer cells, the exact mechanisms of probiotic action in cancer cell death pathways remain unknown. Therefore, we have reported the anti-proliferation activity of isolated *L. fermentum *Ab.RS22 from the traditional dairy on HeLa human cervical cancer cells (normal cells) at molecular and cellular levels (21). The expression of mediated molecules in cancer cell progression and apoptosis including *caspase-3* and *P53*/*PTEN*/*AKT* signaling cascades determine their proliferation and metabolism (22). The association between the activation of the *AKT* signaling pathway and down-regulation of *PTEN* has been observed in cancer cells (23); therefore, the modulation of *PTEN*/*AKT* axis levels in cancer cells through induced P53-apoptotic cell death has crucial roles in the controlling of tumor aggressiveness (24). Recently, researchers have focused on determining the beneficial effects of various probiotics on molecules involved in cancer cell death. For instance, Rahbar Saadat *et al*. reported that *Lactobacillus paracasei* has significant cytotoxicity effects on the SW-480, HT-29, and HCT-116 colon cancer cells. Results from the cytotoxicity assay showed that *L. paracasei* significantly reduced the viability of colon cancer cells at 15 µg/ml (IC_50_ value) at 24 hr. Moreover, findings of the molecular assessments of the study confirmed anticancer activity through simultaneous effect in up-regulation of *BAX* and *caspase-3* genes and down-regulation of *Jak-1*, *Akt1*, and *mTOR *genes (25). Inconsistent with these findings, the present study reported that conditional media of *L. fermentum *Ab.RS22 in IC_50_ values at 24 hr significantly could reduce cell viability of HeLa cells (Figure 2). 

Another study assessed the pre-apoptotic activity of isolated *Leuconostoc mesenteroides* from traditional dairy products on HT-29 cells (colon cancer) at 24 hr. Increased mRNA expression of *Bax*, *caspase-3*, and *MAPK1* genes along with down-regulation of some anti-apoptotic molecules including *Bcl-XL*, *AKT*, and *NF-kB* was confirmed by increased percentage of apoptotic cells; which was detected by flow cytometry (26). In addition, the results of Sungur *et al*. indicated that exopolysaccharides or EPSs of *L. gasseri* strains strongly have anti-proliferative effects on HeLa cells via up-regulation of *Bax* and *caspase-3* genes (27). According to Figure 4, *L. fermentum *Ab.RS22*,* by up-regulation of *Caspase- 3*/*P53* and *PTEN* genes, and also down-regulation of *Akt* gene has pre-apoptotic effects on HeLa cells in IC_50_ value at 24 hr. These findings were confirmed by increased numbers of apoptotic and necrotic cells detected by FITC-Annexin/Pi staining (Figure 3). 

## Conclusion

 One of the most important molecular pathways involved in cell death is apoptosis. The findings of the current study demonstrated that isolated *L. fermentum *Ab.RS22 strains from traditional dairy products have potential anti-proliferation effects on HeLa cancer cells through increased mRNA expression of *caspase-3*, *P53*, and *PTEN* genes, and also decreased mRNA expression of the *Akt* gene. These molecular reports were confirmed through the results of cellular apoptosis assessments by ﬂow cytometry. Overall, our study suggested that isolated *L. fermentum *Ab.RS22 from dairy products is a promising candidate for cancer therapy in cervical cancer. However, further examination of the tumor xenograft model is needed for its usage in clinical trial approaches. 

## Authors’ Contributions

All authors conceived this study and contributed equally to this work: A AF, M S, and P H participated in laboratory tests, data collection, drafting the document, and creating the Figures. H P, A P, and D I mentor contributed to the supervision of the entire work. All authors read and approved the final manuscript.

## Ethical Approval

Not applicable.

## Conflicts of Interest

The authors declare that they have no conflicts of interest.
